# Critical Role of RPS4X in Modulating SCF Complex Formation and Cell Survival

**DOI:** 10.3390/biom15101350

**Published:** 2025-09-23

**Authors:** Satsuki Ryu, Min Ji Kim, Shuya Bando, Yuka Tanaka, Risa Mukai, Yasuhiro Ishihara, Takashi Tominaga, Takayuki Ohshima

**Affiliations:** 1 Faculty of Pharmaceutical Science at Kagawa Campus, Tokushima Bunri University, Takamatsu 760-8542, Kagawa, Japan; s198042@stu.bunri-u.ac.jp (S.R.); s208015@stu.bunri-u.ac.jp (M.J.K.); tominagat@kph.bunri-u.ac.jp (T.T.); 2 Faculty of Science and Engineering, Tokushima Bunri University, Takamatsu 760-8542, Kagawa, Japan; s215622@stu.bunri-u.ac.jp (S.B.); s240381@stu.bunri-u.ac.jp (Y.T.); 3 Department of Cell Biology and Molecular Medicine, Rutgers New Jersey Medical School, Newark, NJ 07103, USA; rm1264@njms.rutgers.edu; 4 Program of Biomedical Science, Graduate School of Integrated Sciences for Life, Hiroshima University, Hiroshima 739-8521, Hiroshima, Japan; ishiyasu@hiroshima-u.ac.jp

**Keywords:** ribosomal proteins, RPS4X, SCF complex, ubiquitination, MCL1, HAX1, apoptosis

## Abstract

Ribosomal proteins have long been recognized as vital components of ribosomes that are involved in protein synthesis. However, emerging evidence indicates that some ribosomal proteins exhibit extraribosomal functions. In this study, we investigated the role of the ribosomal protein S4 X-linked (RPS4X) in the regulation of the Skp1–Cullin1–F-box (SCF) ubiquitin ligase complex and apoptosis. We found that RPS4X expression interfered with SCF complex formation by disrupting the interaction between Cullin1 and Skp1. This disruption suppressed ubiquitination of multiple SCF complex substrates, including the anti-apoptotic proteins myeloid cell leukemia 1 (MCL1) and HS1-associated protein X1 (HAX1). Stabilization of MCL1 and HAX1 by RPS4X led to increased resistance of HeLa cells to doxorubicin-induced apoptosis. These findings suggest that RPS4X contributes to the regulation of protein homeostasis and apoptotic pathways by modulating SCF complex activity, providing new insights into the extraribosomal roles of ribosomal proteins.

## 1. Introduction

The ubiquitin–proteasome system (UPS) maintains cellular protein homeostasis by targeting key regulatory proteins for degradation. One UPS component is the Skp1–Cullin1–F-box (SCF) complex, a well-characterized E3 ubiquitin ligase that ubiquitinates substrates involved in critical processes such as cell cycle progression, signal transduction, and apoptosis [1]. However, despite its importance, the regulatory mechanisms of SCF complex activity remain unclear.

Ribosomal proteins (RPs) play an essential role in ribosome assembly and translation. However, recent studies have revealed that several RPs exhibit extraribosomal functions, including apoptosis regulation, DNA repair, and stress responses [2]. Notably, ribosomal protein L11 (RPL11) and ribosomal protein L22 (RPL22) upregulate the p53 pathway by inhibiting mouse double minute 2 (MDM2)-mediated p53 degradation [3,4]. These findings highlight the broader functional diversity of RPs, beyond protein synthesis.

Ribosomal protein S4 X-linked (RPS4X) is a Cullin1-interacting protein that suppresses the ubiquitination of MDM2, suggesting a potential regulatory effect on SCF complex-mediated ubiquitination [5]. Although RPS4X is implicated in tumor progression in several cancers [6,7], its extraribosomal functions remain poorly characterized, and it is unknown whether RPS4X affects the ubiquitination of other substrates of the SCF complex.

In this study, we investigated whether RPS4X regulates the ubiquitination and stability of additional substrates of the SCF complex. Given the critical role of the UPS in controlling apoptotic regulators, we focused on the anti-apoptotic proteins myeloid cell leukemia 1 (MCL1) and HS1-associated protein X1 (HAX1). MCL1, a BCL-2 family member, inhibits apoptosis by sequestering pro-apoptotic factors [8], whereas HAX1 is critical for maintaining inner mitochondrial membrane potential and induces cell survival [9,10,11,12,13]. Dysregulation of these proteins is associated with resistance to apoptosis and tumor progression. However, it remains unclear whether RPS4X participates in these pathways by stabilizing MCL1 and HAX1. Thus, this study aimed to investigate how RPS4X influences the ubiquitination and stability of the SCF complex substrates and to elucidate the molecular mechanisms by which RPS4X regulates SCF complex assembly and apoptotic signaling.

## 2. Materials and Methods

### 2.1. Cells and Culture

HEK-293T (CRL-3216) and HeLa (CCL-2) cells were purchased from the American Type Culture Collection (ATCC, Baltimore, MD, USA) and maintained in Dulbecco’s modified Eagle’s medium (DMEM; FUJIFILM Wako Pure Chemical, Tokyo, Japan), supplemented with 10% fetal bovine serum (FBS; Biowest, Nuaillé, France), 100 U/mL penicillin, and 100 µg/mL streptomycin at 37 °C under 5% CO_2_ atmosphere.

### 2.2. Antibodies

The antibodies used in this study were anti-HA-horseradish peroxidase (HRP)-linked (3F10; Roche Diagnostics, Indianapolis, IN, USA), anti-Myc (9E10; Santa Cruz Biotechnology, Santa Cruz, CA, USA), anti-FLAG (M2; Sigma-Aldrich, Inc., St. Louis, MO, USA), and anti-green fluorescent protein (GFP)-HRP (HRP-66002; Proteintech, Rosemont, IL, USA) antibodies. Anti-DDDK-tag mAb-HRP-DirecT, anti-Myc-tag mAb-HRP-DirecT, anti-HA-tag mAb-HRP-DirecT, and anti-V5-tag mAb-HRP-DirecT were purchased from Medical & Biological Laboratories (MBL), Nagoya, Japan. Mouse anti-β-actin (MBL) antibody, anti-PARP-1 antibody (B-10; Santa Cruz Biotechnology), and anti-caspase-9 antibody (5B4; MBL) were obtained.

### 2.3. Plasmid Construction

The human β-catenin and human β-TrCP cDNAs were isolated from testicular total RNA by reverse transcription-PCR. β-catenin and β-TrCP were subcloned into the BamHI sites of pcDNA3 containing 3× FLAG, 6× Myc, or 5× HA epitope tags at the N-terminus. The expression vectors for RPS4X, MCL1, HAX1, p21, Cullin1, S-phase kinase-associated protein 1 (Skp1), RING-box protein 1 (Rbx1), FBXO25, and HA-tagged ubiquitin have been previously described [5,14,15,16,17]. The following primer sequences were used:

- β-catenin, 5′-AAAGGATCCATGGCTACTCAAGCTGATTTGAT-3′ (forward), 5′-AAAGGATCCTTACAGGTCAGTATCAAACCAGG -3′ (reverse);
- β-TrCP, 5′-AAAGGATCCATGGACCCGGCCGAGGCGGTGCTGC-3′ (forward), 5′-AAAGGATCCTTATCTGGAGATGTAGGTGTATGTTC-3′ (reverse).

Underlined letters in primers denote restriction sites.

### 2.4. Immunoprecipitation and Immunoblot Analysis

Plasmid transfection into cells was performed as described previously [5,14]. Immunoprecipitation and immunoblot analysis were performed essentially as described previously [5,14,15,16,17].

### 2.5. Transfection Conditions

For co-transfection, 3 µg of plasmid expressing RPS4X and 1 µg of each of the other plasmids were used. Exceptions were Figure 2B (0.5 µg of plasmid expressing HA-Ub), Figure 3A,B (2 µg of plasmid expressing RPS4X), and Figure 3D,E (3 µg of plasmid expressing RPS4X and 0.5 µg of each of the other plasmids). All transfections were performed in HEK293T cells.

### 2.6. In Vivo Ubiquitination Assay

HEK-293T cells were transfected with the indicated plasmids. After 24 h, the cells were treated with 40 µM of MG132, the proteasome inhibitor (Peptide Institute Inc., Osaka, Japan), for 16 h. The cells were lysed in lysis buffer, containing 25 mM Tris-HCl (pH 8.0), 150 mM NaCl, 1 mM ethylene diamine tetra acetic acid [EDTA], 1% Nonidet P-40, 0.1% sodium dodecyl sulfate [SDS], 0.5% deoxycholic acid sodium salt monohydrate [Nacalaitesque, Kyoto, Japan], Pefabloc SC Plus inhibitor [Roche Diagnostics], and cOmplete ULTRA tablets [Roche Diagnostics]. After centrifugation, the supernatants were incubated with suitable antibodies for at least 1 h. Immune complexes were recovered by Protein G-Sepharose^TM^ 4 Fast Flow beads (Cytiva, Shinjuku, Japan) for 1 h. The beads were washed thrice with lysis buffer, and bound proteins were detected by immunoblot analysis.

### 2.7. Cycloheximide Chase Analysis

HEK-293T cells were seeded in 12-well plates (2 × 10^5^ cells/well) and the appropriate expression plasmids were transfected. After 24 h, the cells were treated with 100 µg/mL cycloheximide (FUJIFILM Wako Pure Chemical) to inhibit protein synthesis and then chased for individual intervals. The collected cells were subjected to SDS-polyacrylamide gel electrophoresis (SDS-PAGE), followed by immunoblot analysis. Band intensities were detected by an LAS 3000 image analyzer (Fujifilm, Tokyo, Japan).

### 2.8. Adenovirus Constructs

A recombinant adenoviral (AdV) vector for overexpression was constructed, propagated, and titrated, as previously described [18]. The pBHG plasmid was co-transfected with the pDC316 shuttle vector (Microbix, Mississauga, ON, Canada) containing the V5/RPS4X sequence into HEK293 cells using polyethyleneimine (PEI MAX, linear, molecular weight 40 kDa; Polysciences, Inc., Warrington, PA, USA).

### 2.9. Viral Infection

HeLa cells were seeded in 12-well plates (1 × 10^5^ cells/well) and infected with AdV vector expressing V5-tagged RPS4X at 15 multiplicity of infection (MOI) under culture in DMEM (FUJIFILM Wako Pure Chemical) supplemented with 5% FBS (Biowest), 100 U/mL penicillin, and 100 µg/mL streptomycin. After 48 h of infection, the cells were treated with 10 μM doxorubicin (FUJIFILM Wako Pure Chemical) to induce apoptosis.

## 3. Results

### 3.1. RPS4X Disrupts SCF Complex Formation by Inhibiting the Cullin1–Skp1 Interaction

**Figure 1:**
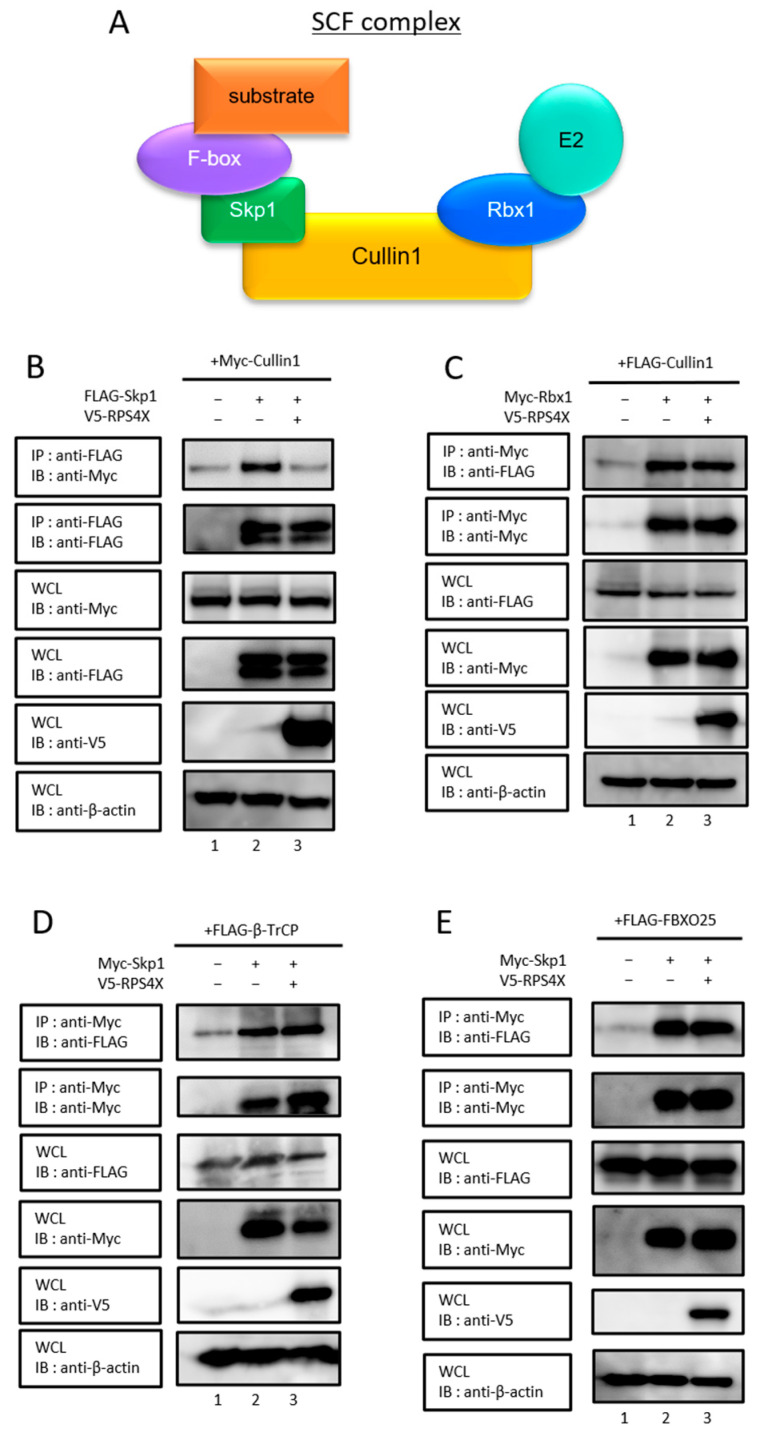
RPS4X interfered with SCF complex formation through specific abrogation of the Cullin1/Skp1 binding. (**A**) Schematic diagram of the SCF complex. The inhibitory effect of RPS4X on SCF complex formations, between Cullin1 and Skp1 (**B**), Cullin1 and Rbx1 (**C**), and the association of Skp1 with F-box proteins, β-TrCP (**D**), and FBXO25 (**E**). After 24 h of transfection, the cells were treated with 40 µM MG132 (a proteasome inhibitor) for 16 h. Cell lysates were immunoprecipitated (IP) using a specific antibody, followed by immunoblotting (IB). Whole-cell lysates (WCLs) were prepared, and the total protein levels were analyzed by IB using appropriate antibodies. RPS4X, ribosomal protein S4X-linked; Skp1, S-phase kinase-associated protein 1; Rbx1, RING-box protein 1.

We previously reported that RPS4X binds to the N-terminal region of Cullin1 [5], a scaffold protein essential for the formation of the SCF complex. The N-terminal domain of Cullin1 serves as the primary interaction site for Skp1, as revealed by crystallographic analysis of the SCF complex [19]. Skp1 functions as an adaptor protein that bridges Cullin1 to F-box proteins, which mediate substrate recognition [20]. The C-terminal domain serves as a binding site for Rbx1, which recruits E2 ubiquitin-conjugating enzymes to the SCF complex (Figure 1A) [19]. Since RPS4X binds to the N-terminal region of Cullin1, we speculated that RPS4X might affect the Cullin1–Skp1 interaction. Indeed, co-expression of RPS4X was markedly suppressed in the interaction between Cullin1 and Skp1 (Figure 1B, lane 2 vs. lane 3, top panel).

To determine whether the inhibitory effect of RPS4X on SCF complex formation is specific to Cullin1–Skp1 binding, we next examined whether RPS4X affects the interactions of the SCF complex with other components. We first assessed the effect of RPS4X on the interaction between Cullin1 and Rbx1. In contrast to its effect on Cullin1–Skp1 binding, RPS4X did not affect the association of Cullin1 with Rbx1 (Figure 1C, lane 2 vs. lane 3, top panel). Next, we examined whether RPS4X influences the association of Skp1 with F-box proteins. We focused on two representative F-box proteins: β-TrCP and FBXO25. β-TrCP is a well-characterized F-box protein that targets several cell cycle- and apoptosis-related substrates, including β-catenin, IκBα, and MCL1, and is frequently implicated in cancer signaling [21,22,23]. FBXO25 regulates transcription factors and apoptosis-related proteins and is considered a biologically relevant F-box protein in multiple cellular contexts [24,25]. To analyze the effect of RPS4X on the interaction between Skp1 and β-TrCP, co-immunoprecipitation was performed. The association of Skp1 with β-TrCP was not affected by RPS4X overexpression (Figure 1D, lane 2 vs. lane 3, top panel). A similar result was obtained for FBXO25 (Figure 1E, lane 2 vs. lane 3, top panel). Collectively, these results indicate that RPS4X specifically interferes with the Cullin1–Skp1 interaction, without affecting the association of other SCF complex components.

### 3.2. RPS4X Suppresses SCF Complex-Mediated Ubiquitination of Its Target Proteins

Skp1, a conserved cytoplasmic and nuclear protein, plays a pivotal role in mediating the assembly of the SCF complex by binding to both Cullin1 and F-box protein, forming a platform for substrate recruitment [20,26]. Given the essential role of Skp1 in bridging the scaffold and substrate receptor modules, disruption of the Skp1–Cullin1 interaction is expected to impair SCF complex function.

**Figure 2:**
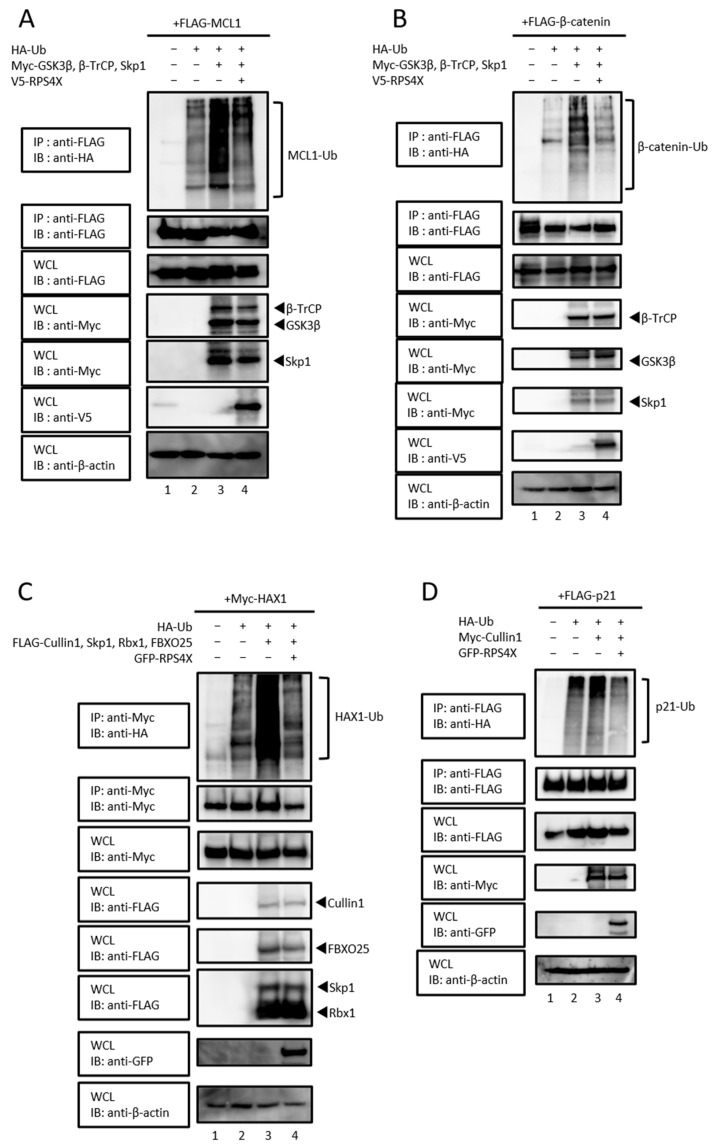
SCF complex-mediated ubiquitination of its target proteins was inhibited by RPS4X. In vivo ubiquitination assays were performed for the following target proteins: MCL1 (**A**), β-catenin (**B**), HAX1 (**C**), and p21 (**D**). Cell lysates were subjected to IP using a specific antibody, followed by IB. WCLs were prepared, and the total protein levels were analyzed by IB using appropriate antibodies. MCL1, myeloid cell leukemia-1; GSK-3β, glycogen synthase kinase-3 beta; HAX1, HS-1-associated protein X-1.

We previously demonstrated that RPS4X suppresses the SCF-dependent ubiquitination of MDM2 [5], raising the possibility that RPS4X negatively regulates SCF complex activity. Based on these observations, we postulated that RPS4X could widely suppress the ubiquitination of the target proteins of the SCF complex, in addition to MDM2. To test this hypothesis, we investigated whether RPS4X regulates the SCF complex-mediated ubiquitination of its target proteins MCL1, β-catenin, HAX1, and p21, which are known to be degraded by SCF^β-TrCP^, SCF^FBXO25^, and SCF^Skp2^, respectively [21,22,23,25,27,28,29]. We performed in vivo ubiquitination assays for the epitope-tagged target proteins FLAG-MCL1 (Figure 2A), FLAG-β-catenin (Figure 2B), Myc-HAX1 (Figure 2C), and FLAG-p21 (Figure 2D) in the presence of the proteasome inhibitor MG132. Glycogen synthase kinase-3β (GSK3β) was co-expressed in assays involving β-catenin (Figure 2B) and MCL1 (Figure 2A), as it is a well-established kinase that phosphorylates these substrates and primes them for SCF^β-TrCP^-mediated ubiquitination [30,31,32].

In each case, co-expression of the SCF complex components promoted polyubiquitination of the respective substrates, which was markedly suppressed by RPS4X expression (Figure 2). These results suggest that RPS4X selectively inhibits the Cullin1–Skp1 interaction, thereby negatively regulating the ubiquitination by the SCF complex.

### 3.3. RPS4X Delays Degradation of the SCF Complex Target Proteins MCL1 and HAX1

RPS4X is frequently overexpressed in various cancers, including colorectal and intrahepatic cholangiocarcinoma, and its high expression is associated with poor patient prognosis [6,7]. These findings raise the possibility that RPS4X contributes to tumor progression through the regulation of cell survival pathways. Among the SCF complex substrates, the anti-apoptotic proteins MCL1 and HAX1 have been shown to play critical roles in promoting cancer cell survival and therapeutic resistance. MCL1, a member of the BCL-2 family, inhibits apoptosis by sequestering pro-apoptotic factors at the mitochondrial outer membrane, and its overexpression has been frequently observed in various human malignancies, contributing to tumor progression and therapeutic resistance [33,34]. HAX1 supports the maintenance of the inner mitochondrial membrane potential and prevents caspase activation, and its dysregulation is associated with poor prognosis in several cancers [9,35]. In the present study, we focused on the stability of MCL1 and HAX1 to investigate the role of RPS4X in cell survival pathways.

**Figure 3:**
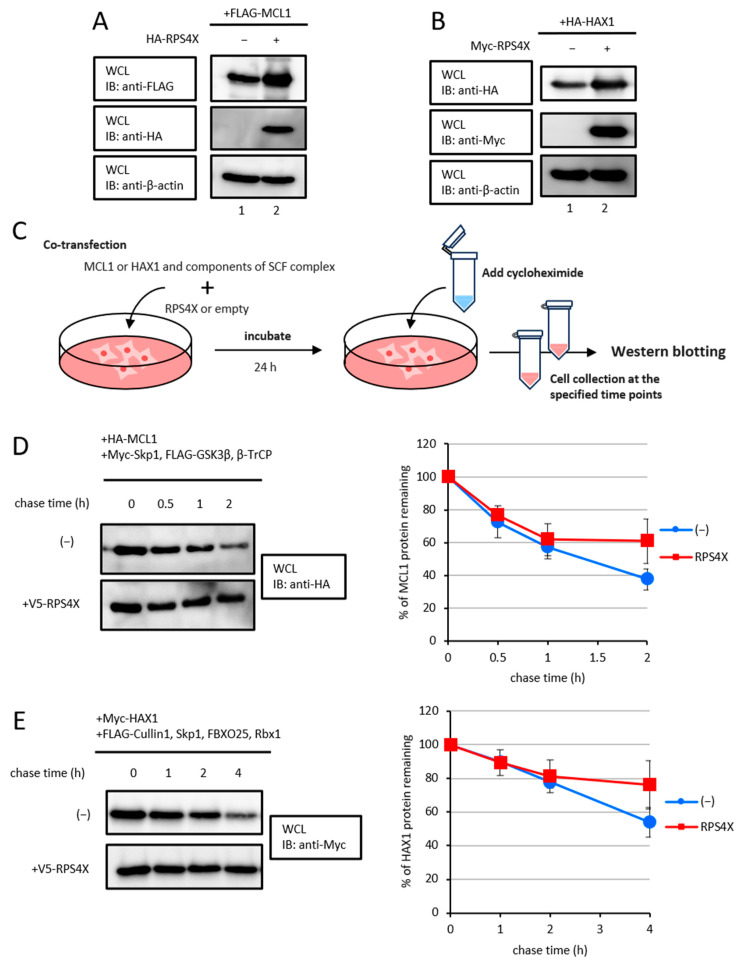
RPS4X enhanced the stability of the SCF complex target proteins. RPS4X increases the steady-state protein levels of MCL1 (**A**) and HAX1 (**B**). The cell extracts were subjected to SDS-PAGE, followed by IB using specific antibodies. (**C**) Experiment protocol for cycloheximide chase analysis. (**D**) MCL1 protein levels were assessed. After 24 h of transfection, the cells were treated with 100 µg/mL cycloheximide and chased for the indicated time intervals. Cell lysates were prepared, and protein levels were analyzed by IB using anti-HA antibody. The intensity of each band from three independent experiments was quantified by densitometry using ImageJ v1.43 software, and the mean values are graphed. Data represent the means ± standard deviation (SD) of three independent experiments. (**E**) HAX1 protein levels were assessed. After 24 h of transfection, the cells were treated with cycloheximide and chased for the indicated time intervals. Cell lysates were prepared, and protein levels were analyzed by IB using anti-Myc antibody. Each band intensity from three independent experiments was quantified by densitometry using ImageJ v1.43 software, and the mean values are graphed. Data represent the means ± SD of three independent experiments.

To examine whether RPS4X increases the steady-state protein levels of MCL1 and HAX1. As shown in Figure 3A,B, RPS4X overexpression resulted in an increase in MCL1 and HAX1 protein levels.

Given that RPS4X increases the steady-state protein levels of MCL1 and HAX1, we subsequently performed cycloheximide chase assays (Figure 3C). For MCL1, the cells were co-expressed with HA-MCL1 and Myc-Skp1, FLAG-GSK3β, and -β-TrCP1, in the presence or absence of V5-RPS4X. After 24 h, the cells were treated with 100 µg/mL cycloheximide to block protein synthesis, and the MCL1 protein levels were analyzed by western blotting (Figure 3D). For HAX1, the cells were co-expressed with Myc-HAX1, FLAG-Cullin1, -Skp1, -FBXO25, and -Rbx1 in the presence or absence of V5-RPS4X. The cells were treated with cycloheximide in the same manner, and time-course western blot analyses were conducted to evaluate HAX1 protein stability (Figure 3E). In both cases, RPS4X expression in the cells significantly prolonged the half-life of the MCL1 and HAX1 proteins compared to that of cells not expressing RPS4X. Collectively, these data show that RPS4X enhances the stability of MCL1 and HAX1 proteins by preventing SCF complex-mediated ubiquitination.

### 3.4. RPS4X Suppresses Doxorubicin-Induced Apoptosis

**Figure 4:**
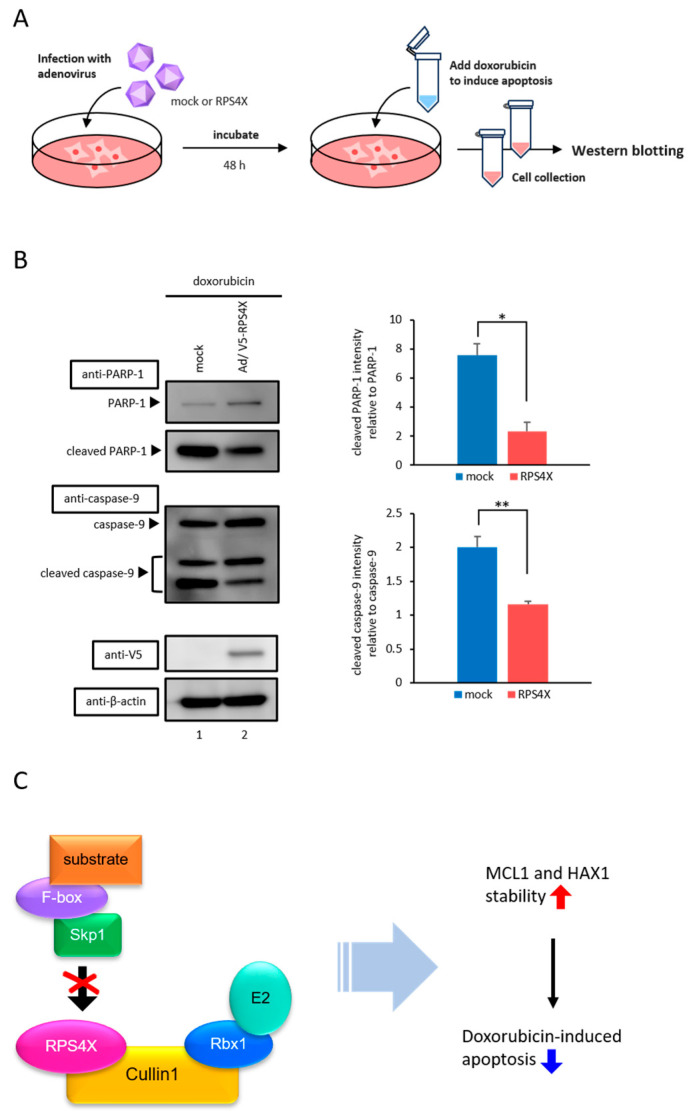
Doxorubicin-induced apoptosis was suppressed by RPS4X. (**A**) Schematic representation of the adenovirus infection assay. (**B**) HeLa cells were infected with adenoviral (AdV) vector expressing V5-tagged RPS4X and treated with doxorubicin, as described in Section 2. WCLs were prepared and subjected to IB with anti-PARP-1, anti-caspase-9, anti-V5, and anti-β-actin antibodies. The bar chart shows the quantification of cleaved PARP-1/PARP-1 and cleaved caspase-9/caspase-9 ratios. Band intensities from three independent experiments were quantified by densitometry using ImageJ v1.43 software. The data represent the means of three independent experiments. Error bars represent SD. * *p*  <  0.05, ** *p*  <  0.01. (**C**) Schematic diagram showing that RPS4X specifically inhibits the binding between Cullin1 and Skp1 and affects the ability of cell survival. PARP-1, poly (ADP-ribose) polymerase 1.

Given that RPS4X stabilizes anti-apoptotic proteins, such as MCL1 and HAX1, we next examined whether RPS4X affects apoptosis. To assess the functional effects of RPS4X expression on apoptosis, cell death was induced using doxorubicin, a chemotherapeutic agent that activates the apoptotic pathway. HeLa cells were transduced with either a mock AdV or an AdV expressing V5-tagged RPS4X (AdV-RPS4X). After 48 h of infection, the cells were treated with 1 µM doxorubicin for 24 h. Following treatment, the cell lysates were collected and subjected to western blot analysis to detect the cleavage of poly (ADP-ribose) polymerase 1 (PARP-1) and caspase-9, which are well-established markers of apoptosis (Figure 4A) [36,37,38]. Anti-PARP-1 and anti-caspase-9 antibodies were used to observe apoptotic activation. As shown in Figure 4B, the cleavage of PARP-1 and caspase-9 was markedly suppressed in cells expressing RPS4X compared to that in mock AdV-infected cells, indicating that RPS4X inhibits doxorubicin-induced apoptosis.

## 4. Discussion

In this study, we identified a novel extraribosomal function of RPS4X as a negative regulator of SCF complex-mediated ubiquitination and apoptotic signaling. Specifically, RPS4X disrupts the assembly of the SCF complex by impairing the Cullin1–Skp1 interaction, leading to the stabilization of anti-apoptotic proteins such as MCL1 and HAX1. This stabilization contributes to apoptotic resistance, highlighting a novel mechanism by which RPs can influence cell fate beyond their well-known role in translation (Figure 4C).

Over the last couple of decades, findings have suggested that RPs contribute to cellular homeostasis beyond their canonical roles in translation. For instance, RPL5 and RPL11 have been shown to regulate ubiquitin-dependent signaling by stabilizing p53 through the inhibition of MDM2 [3,39]. Our results indicate that RPS4X acts more broadly by interfering with the assembly of the SCF ubiquitin ligase complex. This suggests that RPs regulate protein degradation pathways, thereby maintaining proteostasis.

Nucleolar stress triggered by ribosomal RNA deficiency, RP imbalance, nutrient starvation, or chemotherapeutic agents leads to the release of specific RPs from the nucleolus into the nucleoplasm [40]. Several studies have demonstrated that the released RPs, such as RPL5, RPL11, RPL23, and RPS7, can exert extraribosomal functions, including the regulation of key stress-response pathways such as cell cycle arrest [39,41,42,43]. These findings suggest that RPs act as stress sensors and induce adaptive cellular responses. Although further studies are required to clarify the biological significance of RPS4X-mediated SCF inhibition, our findings raise an interesting possibility. One plausible hypothesis is that under nucleolar or genotoxic stress, suppression of the SCF complex activity by RPS4X could stabilize anti-apoptotic proteins such as MCL1 and HAX1, thereby enhancing cell survival. However, abnormal RPS4X expression may have pathological consequences. For instance, in colorectal cancer, RPS4X overexpression has been associated with poor prognosis and increased metastasis [6], suggesting that the stabilization of survival factors may contribute to tumor progression. Because nucleolar stress is frequently observed in rapidly growing tumor cells, it is possible that cancer cells exploit the function of RPS4X to overcome these stressful conditions. Thus, RPS4X may act as a double-edged sword, promoting cellular resilience under stress and potentially facilitating oncogenic processes when dysregulated.

Our findings suggest that RPS4X contributes to tumor progression by stabilizing anti-apoptotic proteins and promoting resistance to cell death. MCL1 and HAX1 are key regulators of apoptotic resistance, and their overexpression is associated with poor prognosis and therapeutic resistance in various cancers [9,33,34,35]. Although a direct relationship between RPS4X and these survival factors has not been established, recent studies have reported elevated RPS4X expression in multiple malignancies. For instance, RPS4X overexpression correlates with poor prognosis in colorectal cancer [6] and intrahepatic cholangiocarcinoma [7]. These observations raise the possibility that tumor cells enhance their resistance to apoptosis and survival through the RPS4X-mediated stabilization of MCL1 and HAX1. In addition, considering that doxorubicin is a key chemotherapeutic agent for several solid and hematological malignancies, our observation that RPS4X suppresses doxorubicin-induced apoptosis implies that RPS4X overexpression may not only promote tumor progression but also contribute to chemotherapeutic resistance. This is consistent with previous studies showing that the stabilization of MCL1 and HAX1 is implicated in drug resistance. For example, activation of β2-adrenoreceptor signaling delays the loss of MCL1 protein expression and increases resistance of prostate cancer xenografts to cytotoxic therapies [44], and inhibition of *HAX-1* by microRNA-125a significantly promotes cisplatin-induced apoptosis in laryngeal cancer stem cells and reduces resistance to various chemotherapeutic agents, including doxorubicin [45]. Given the growing interest in developing MCL1-targeted therapies for cancers, such as acute myeloid leukemia and breast cancer [46], RPS4X could represent an upstream regulatory factor worth targeting to overcome apoptotic resistance. Future studies examining the co-expression of RPS4X, MCL1, and HAX1 in clinical tumor samples, as well as functional studies in patient-derived models, will be essential to validate the potential oncogenic role of RPS4X.

## 5. Conclusions

Our study expands the understanding of ribosomal protein functions through the involvement of RPS4X in ubiquitin signaling and apoptosis regulation. A deeper investigation of these mechanisms may offer new therapeutic strategies for overcoming apoptosis resistance in cancer.

## Data Availability

The data that support the findings of this study are available from the corresponding author upon reasonable request. All original images are available in the Supplementary Materials.

## Supplementary Materials

The following supporting information can be downloaded at https://www.mdpi.com/article/10.3390/biom15101350/s1. Original Western Blots.
