# Supplementary figures and images for: Critical Role of RPS4X in Modulating SCF Complex Formation and Cell Survival
Source: Biomolecules. 2025 Sep 23;15(10):1350. doi: 10.3390/biom15101350 (PMC12561996; doi:10.3390/biom15101350)

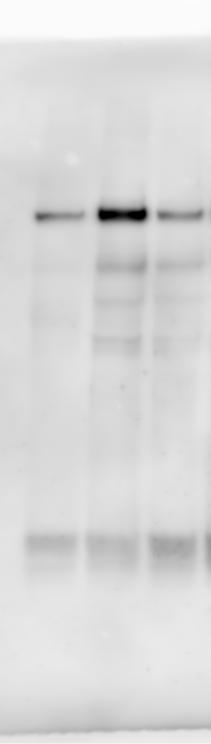

Supplement: Supplementary file 1 [file biomolecules-15-01350-s001.zip › biomolecules-3838184-original-images/Fig.1/Fig.1A. 1st panel.tif]

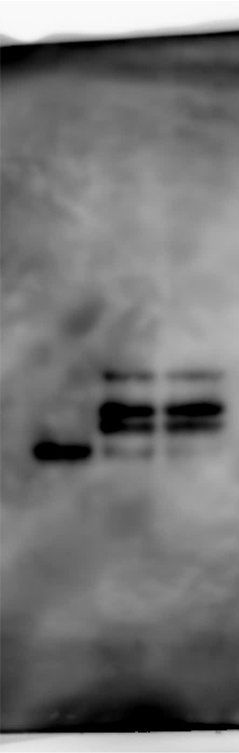

Supplement: Supplementary file 1 [file biomolecules-15-01350-s001.zip › biomolecules-3838184-original-images/Fig.1/Fig.1A. 2nd panel.tif]

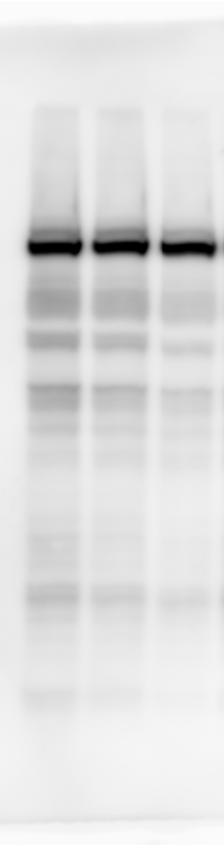

Supplement: Supplementary file 1 [file biomolecules-15-01350-s001.zip › biomolecules-3838184-original-images/Fig.1/Fig.1A. 3rd panel.tif]

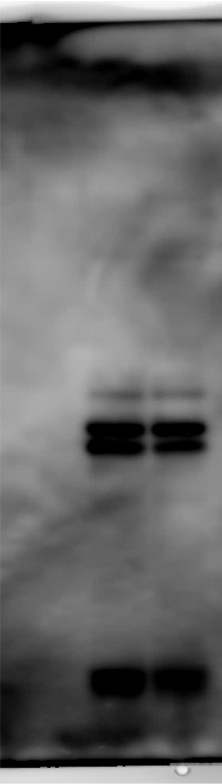

Supplement: Supplementary file 1 [file biomolecules-15-01350-s001.zip › biomolecules-3838184-original-images/Fig.1/Fig.1A. 4th panel.tif]

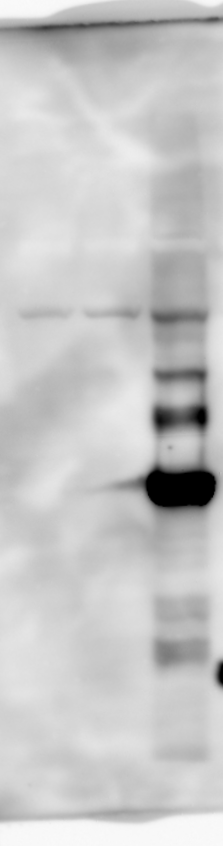

Supplement: Supplementary file 1 [file biomolecules-15-01350-s001.zip › biomolecules-3838184-original-images/Fig.1/Fig.1A. 5th panel.tif]

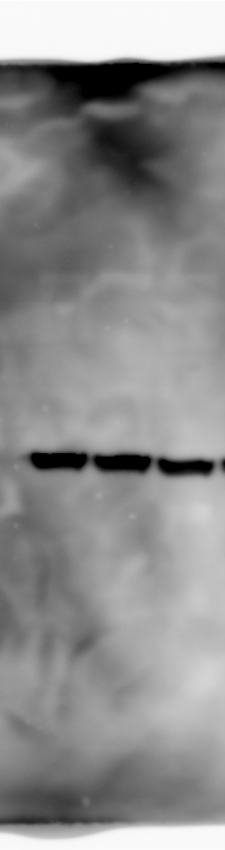

Supplement: Supplementary file 1 [file biomolecules-15-01350-s001.zip › biomolecules-3838184-original-images/Fig.1/Fig.1A. 6th panel.tif]

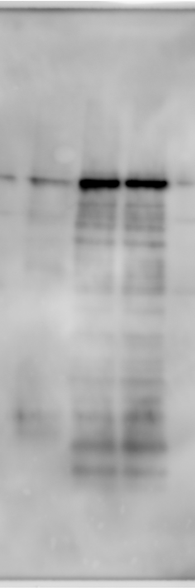

Supplement: Supplementary file 1 [file biomolecules-15-01350-s001.zip › biomolecules-3838184-original-images/Fig.1/Fig.1B. 1st panel.tif]

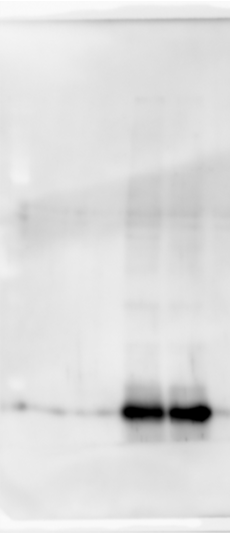

Supplement: Supplementary file 1 [file biomolecules-15-01350-s001.zip › biomolecules-3838184-original-images/Fig.1/Fig.1B. 2nd panel.tif]

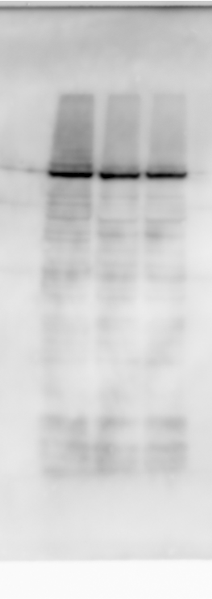

Supplement: Supplementary file 1 [file biomolecules-15-01350-s001.zip › biomolecules-3838184-original-images/Fig.1/Fig.1B. 3rd panel.tif]

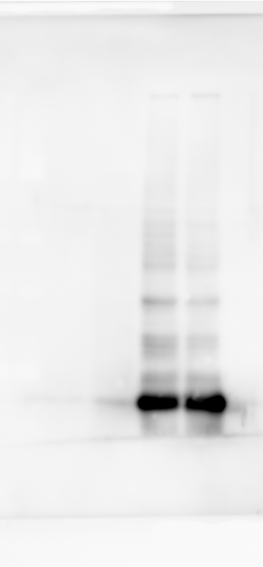

Supplement: Supplementary file 1 [file biomolecules-15-01350-s001.zip › biomolecules-3838184-original-images/Fig.1/Fig.1B. 4th panel.tif]

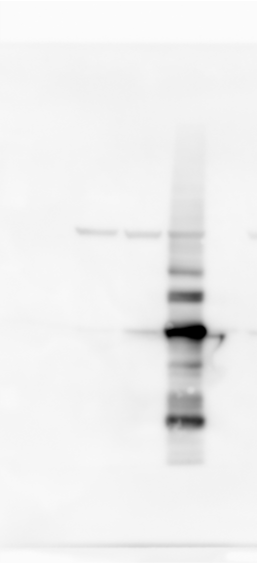

Supplement: Supplementary file 1 [file biomolecules-15-01350-s001.zip › biomolecules-3838184-original-images/Fig.1/Fig.1B. 5th panel.tif]

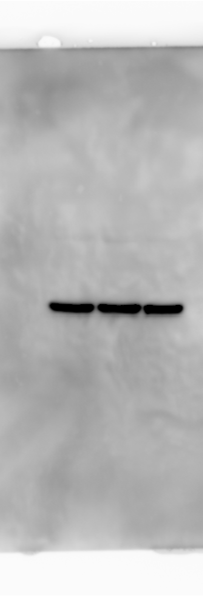

Supplement: Supplementary file 1 [file biomolecules-15-01350-s001.zip › biomolecules-3838184-original-images/Fig.1/Fig.1B. 6th panel.tif]

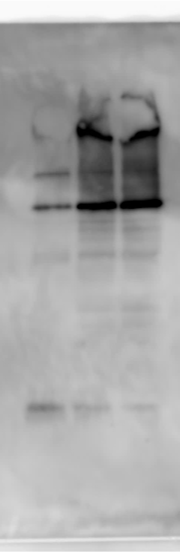

Supplement: Supplementary file 1 [file biomolecules-15-01350-s001.zip › biomolecules-3838184-original-images/Fig.1/Fig.1C. 1st panel.tif]

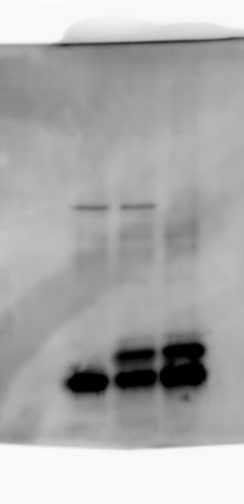

Supplement: Supplementary file 1 [file biomolecules-15-01350-s001.zip › biomolecules-3838184-original-images/Fig.1/Fig.1C. 2nd panel.tif]

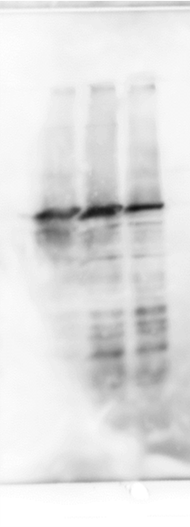

Supplement: Supplementary file 1 [file biomolecules-15-01350-s001.zip › biomolecules-3838184-original-images/Fig.1/Fig.1C. 3rd panel.tif]

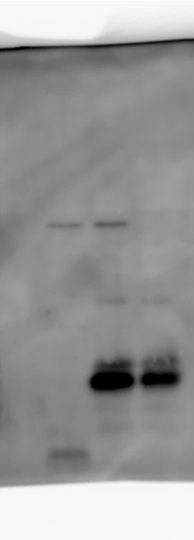

Supplement: Supplementary file 1 [file biomolecules-15-01350-s001.zip › biomolecules-3838184-original-images/Fig.1/Fig.1C. 4th panel.tif]

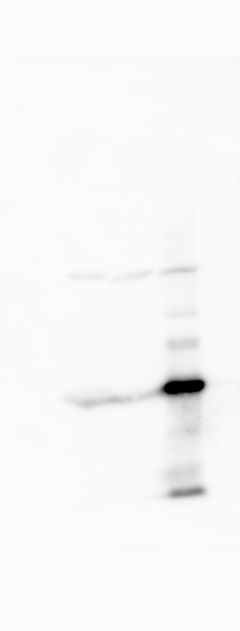

Supplement: Supplementary file 1 [file biomolecules-15-01350-s001.zip › biomolecules-3838184-original-images/Fig.1/Fig.1C. 5th panel.tif]

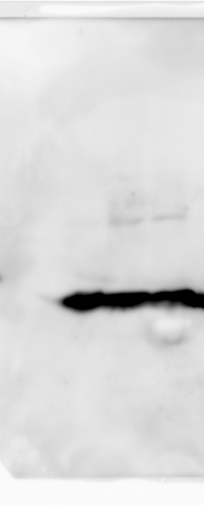

Supplement: Supplementary file 1 [file biomolecules-15-01350-s001.zip › biomolecules-3838184-original-images/Fig.1/Fig.1C. 6th panel.tif]

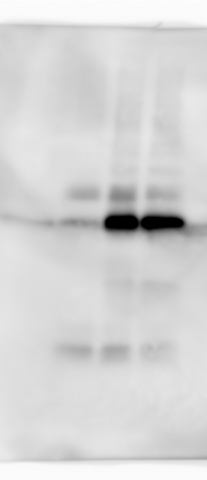

Supplement: Supplementary file 1 [file biomolecules-15-01350-s001.zip › biomolecules-3838184-original-images/Fig.1/Fig.1D. 1st panel.tif]

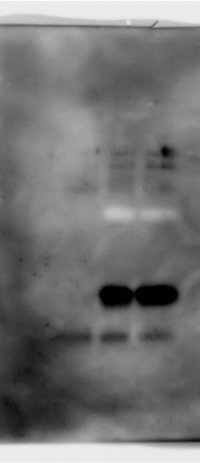

Supplement: Supplementary file 1 [file biomolecules-15-01350-s001.zip › biomolecules-3838184-original-images/Fig.1/Fig.1D. 2nd panel.tif]

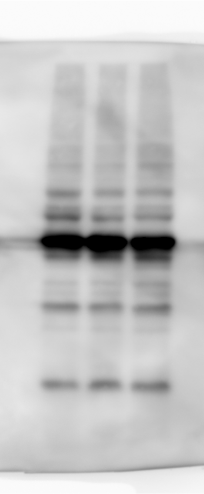

Supplement: Supplementary file 1 [file biomolecules-15-01350-s001.zip › biomolecules-3838184-original-images/Fig.1/Fig.1D. 3rd panel.tif]

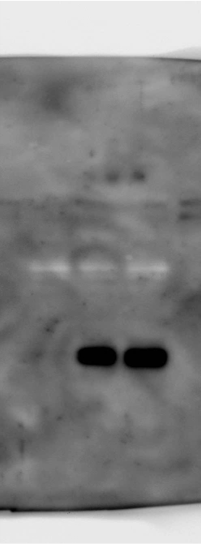

Supplement: Supplementary file 1 [file biomolecules-15-01350-s001.zip › biomolecules-3838184-original-images/Fig.1/Fig.1D. 4th panel.tif]

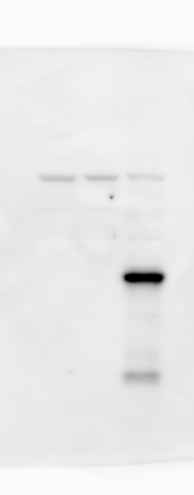

Supplement: Supplementary file 1 [file biomolecules-15-01350-s001.zip › biomolecules-3838184-original-images/Fig.1/Fig.1D. 5th panel.tif]

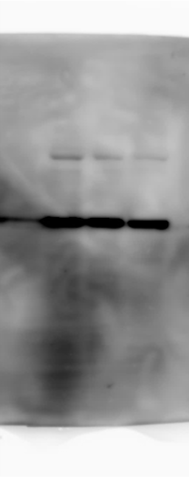

Supplement: Supplementary file 1 [file biomolecules-15-01350-s001.zip › biomolecules-3838184-original-images/Fig.1/Fig.1D. 6th panel.tif]

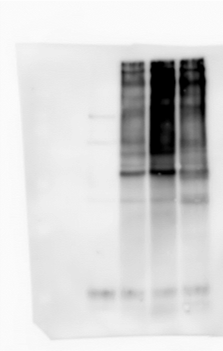

Supplement: Supplementary file 1 [file biomolecules-15-01350-s001.zip › biomolecules-3838184-original-images/Fig.2/Fig.2A. 1st panel.tif]

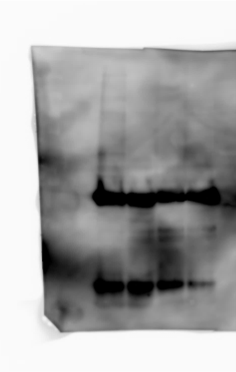

Supplement: Supplementary file 1 [file biomolecules-15-01350-s001.zip › biomolecules-3838184-original-images/Fig.2/Fig.2A. 2nd panel.tif]

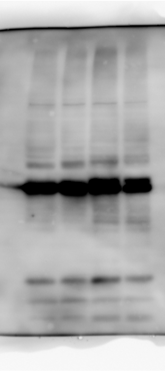

Supplement: Supplementary file 1 [file biomolecules-15-01350-s001.zip › biomolecules-3838184-original-images/Fig.2/Fig.2A. 3rd panel.tif]

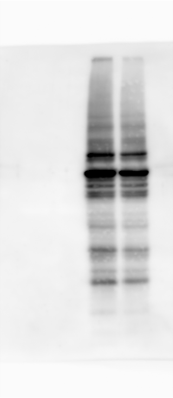

Supplement: Supplementary file 1 [file biomolecules-15-01350-s001.zip › biomolecules-3838184-original-images/Fig.2/Fig.2A. 4th panel.tif]

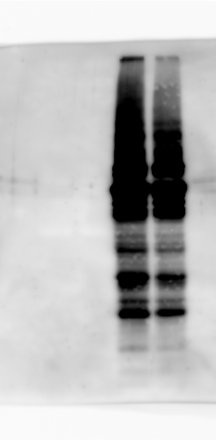

Supplement: Supplementary file 1 [file biomolecules-15-01350-s001.zip › biomolecules-3838184-original-images/Fig.2/Fig.2A. 5th panel.tif]

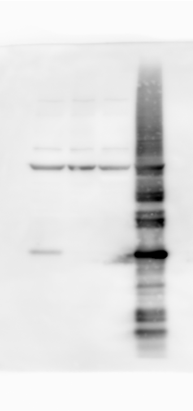

Supplement: Supplementary file 1 [file biomolecules-15-01350-s001.zip › biomolecules-3838184-original-images/Fig.2/Fig.2A. 6th panel.tif]

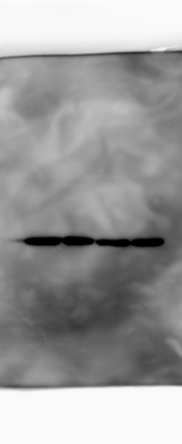

Supplement: Supplementary file 1 [file biomolecules-15-01350-s001.zip › biomolecules-3838184-original-images/Fig.2/Fig.2A. 7th panel.tif]

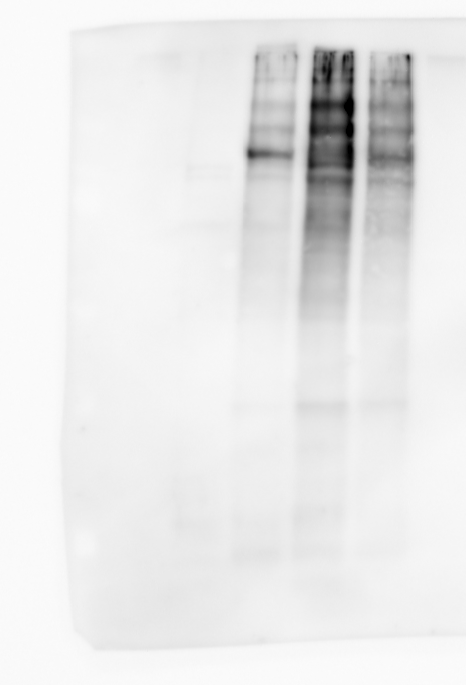

Supplement: Supplementary file 1 [file biomolecules-15-01350-s001.zip › biomolecules-3838184-original-images/Fig.2/Fig.2B. 1st panel.tif]

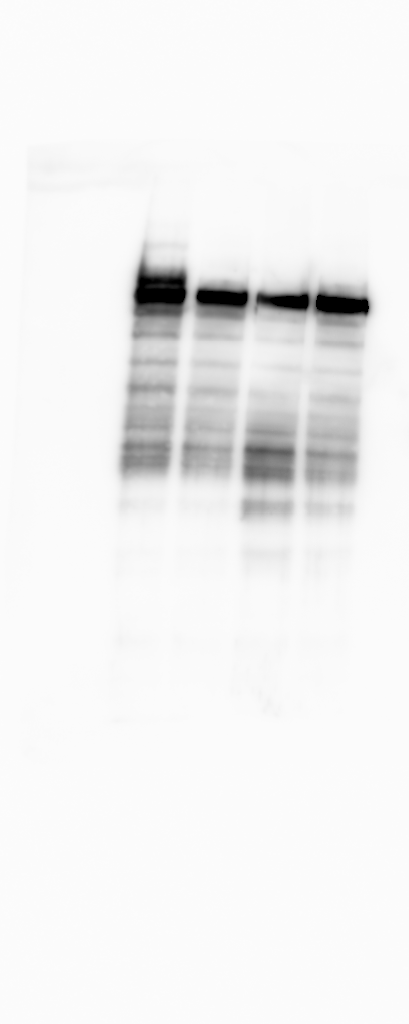

Supplement: Supplementary file 1 [file biomolecules-15-01350-s001.zip › biomolecules-3838184-original-images/Fig.2/Fig.2B. 2nd panel.tif]

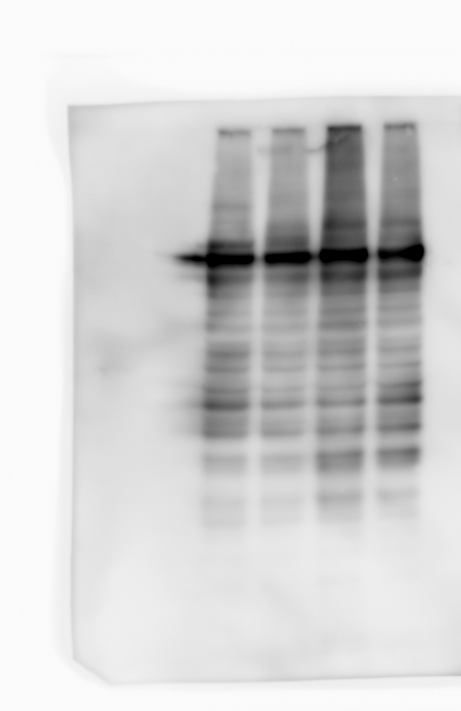

Supplement: Supplementary file 1 [file biomolecules-15-01350-s001.zip › biomolecules-3838184-original-images/Fig.2/Fig.2B. 3rd panel.tif]

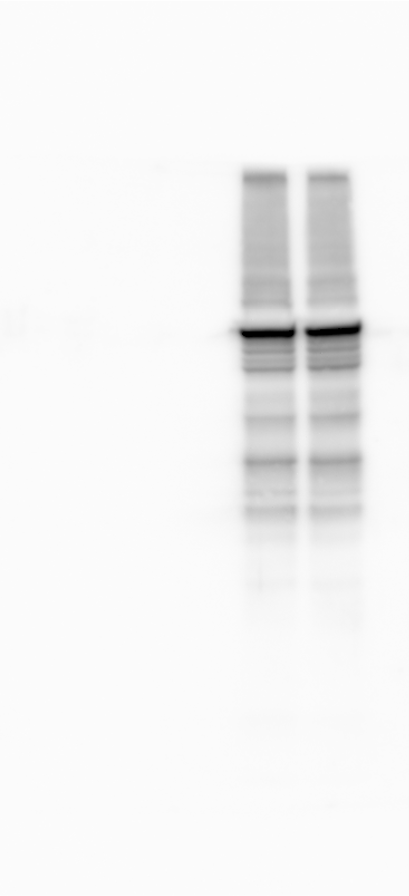

Supplement: Supplementary file 1 [file biomolecules-15-01350-s001.zip › biomolecules-3838184-original-images/Fig.2/Fig.2B. 4th panel.tif]

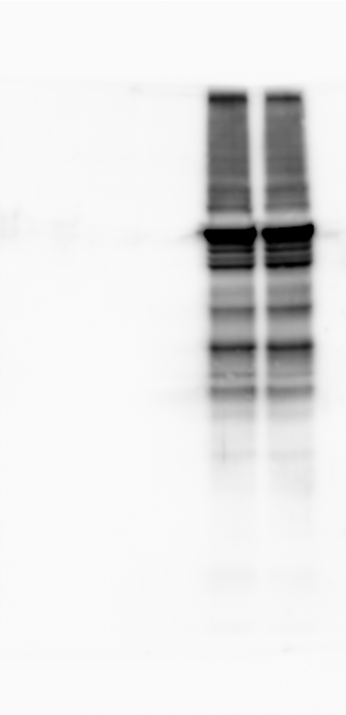

Supplement: Supplementary file 1 [file biomolecules-15-01350-s001.zip › biomolecules-3838184-original-images/Fig.2/Fig.2B. 5th panel.tif]

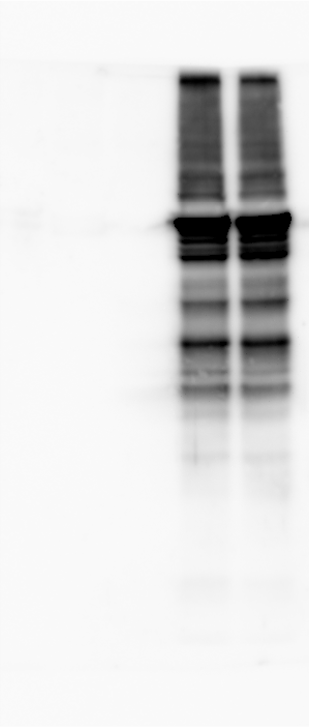

Supplement: Supplementary file 1 [file biomolecules-15-01350-s001.zip › biomolecules-3838184-original-images/Fig.2/Fig.2B. 6th panel.tif]

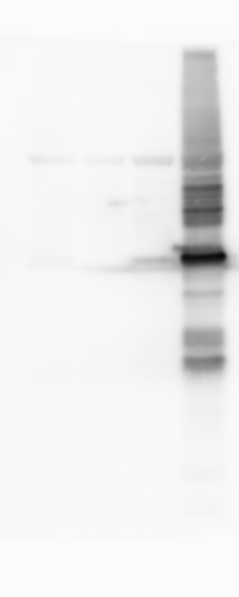

Supplement: Supplementary file 1 [file biomolecules-15-01350-s001.zip › biomolecules-3838184-original-images/Fig.2/Fig.2B. 7th panel.tif]

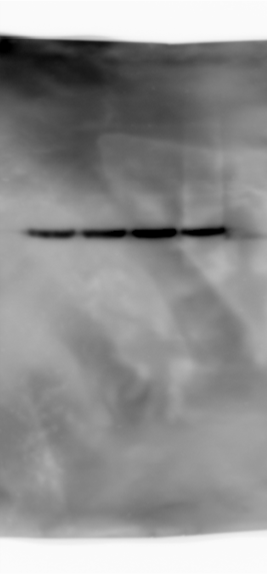

Supplement: Supplementary file 1 [file biomolecules-15-01350-s001.zip › biomolecules-3838184-original-images/Fig.2/Fig.2B. 8th panel.tif]

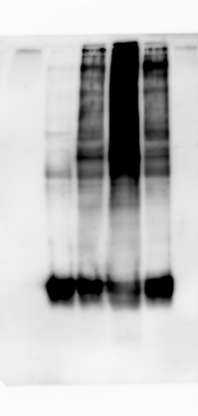

Supplement: Supplementary file 1 [file biomolecules-15-01350-s001.zip › biomolecules-3838184-original-images/Fig.2/Fig.2C. 1st panel.tif]

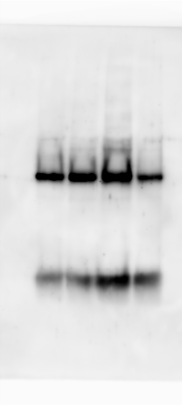

Supplement: Supplementary file 1 [file biomolecules-15-01350-s001.zip › biomolecules-3838184-original-images/Fig.2/Fig.2C. 2nd panel.tif]

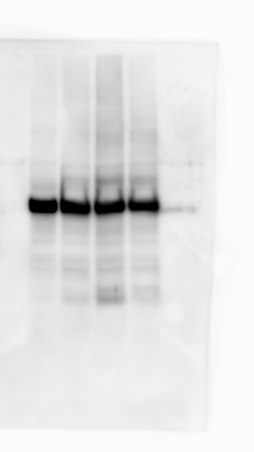

Supplement: Supplementary file 1 [file biomolecules-15-01350-s001.zip › biomolecules-3838184-original-images/Fig.2/Fig.2C. 3rd panel.tif]

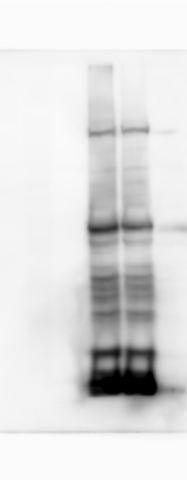

Supplement: Supplementary file 1 [file biomolecules-15-01350-s001.zip › biomolecules-3838184-original-images/Fig.2/Fig.2C. 4, 5, and 6th panel.tif]

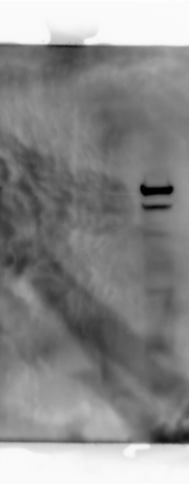

Supplement: Supplementary file 1 [file biomolecules-15-01350-s001.zip › biomolecules-3838184-original-images/Fig.2/Fig.2C. 7th panel.tif]

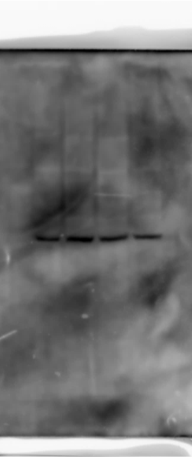

Supplement: Supplementary file 1 [file biomolecules-15-01350-s001.zip › biomolecules-3838184-original-images/Fig.2/Fig.2C. 8th panel.png]

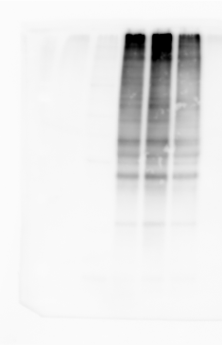

Supplement: Supplementary file 1 [file biomolecules-15-01350-s001.zip › biomolecules-3838184-original-images/Fig.2/Fig.2D. 1st panel.tif]

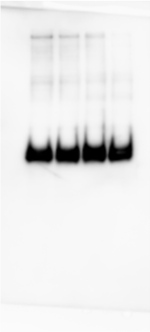

Supplement: Supplementary file 1 [file biomolecules-15-01350-s001.zip › biomolecules-3838184-original-images/Fig.2/Fig.2D. 2nd panel.tif]

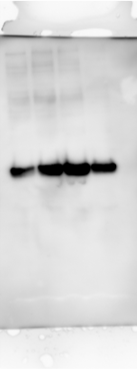

Supplement: Supplementary file 1 [file biomolecules-15-01350-s001.zip › biomolecules-3838184-original-images/Fig.2/Fig.2D. 3rd panel.tif]

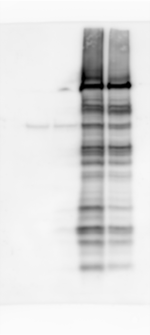

Supplement: Supplementary file 1 [file biomolecules-15-01350-s001.zip › biomolecules-3838184-original-images/Fig.2/Fig.2D. 4th panel.tif]

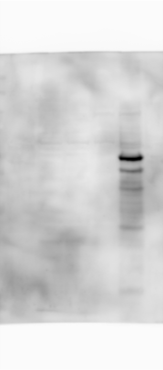

Supplement: Supplementary file 1 [file biomolecules-15-01350-s001.zip › biomolecules-3838184-original-images/Fig.2/Fig.2D. 5th panel.tif]

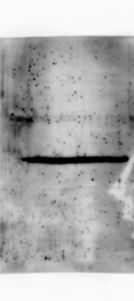

Supplement: Supplementary file 1 [file biomolecules-15-01350-s001.zip › biomolecules-3838184-original-images/Fig.2/Fig.2D. 6th panel.tif]

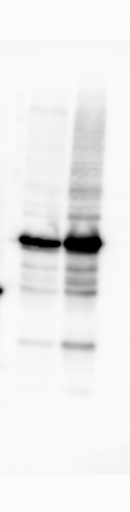

Supplement: Supplementary file 1 [file biomolecules-15-01350-s001.zip › biomolecules-3838184-original-images/Fig.3/Fig.3A. 1st panel.tif]

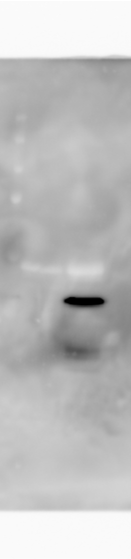

Supplement: Supplementary file 1 [file biomolecules-15-01350-s001.zip › biomolecules-3838184-original-images/Fig.3/Fig.3A. 2nd panel.tif]

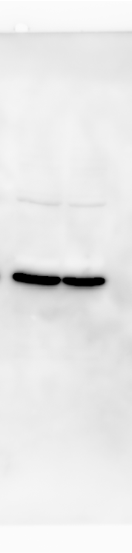

Supplement: Supplementary file 1 [file biomolecules-15-01350-s001.zip › biomolecules-3838184-original-images/Fig.3/Fig.3A. 3rd panel.tif]

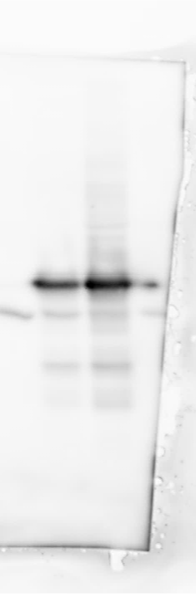

Supplement: Supplementary file 1 [file biomolecules-15-01350-s001.zip › biomolecules-3838184-original-images/Fig.3/Fig.3B. 1st panel.tif]

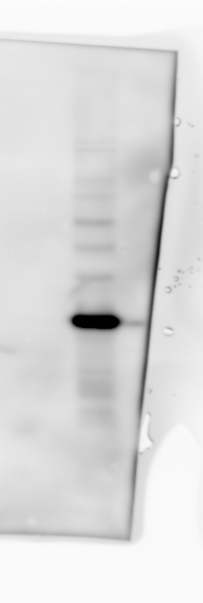

Supplement: Supplementary file 1 [file biomolecules-15-01350-s001.zip › biomolecules-3838184-original-images/Fig.3/Fig.3B. 2nd panel.tif]

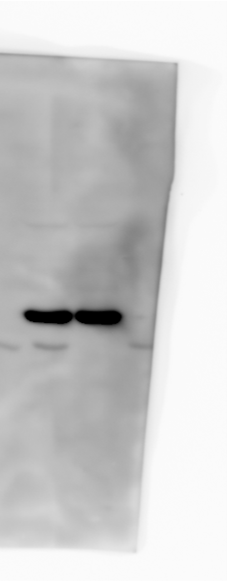

Supplement: Supplementary file 1 [file biomolecules-15-01350-s001.zip › biomolecules-3838184-original-images/Fig.3/Fig.3B. 3rd panel.tif]

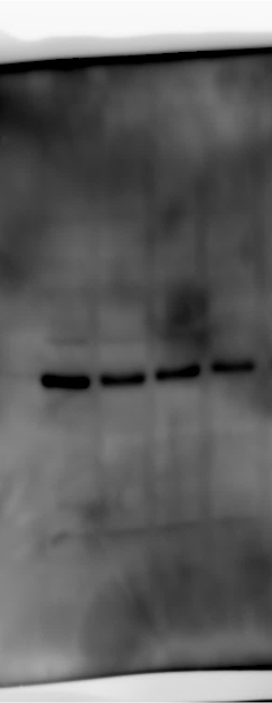

Supplement: Supplementary file 1 [file biomolecules-15-01350-s001.zip › biomolecules-3838184-original-images/Fig.3/Fig.3C. lower panel.tif]

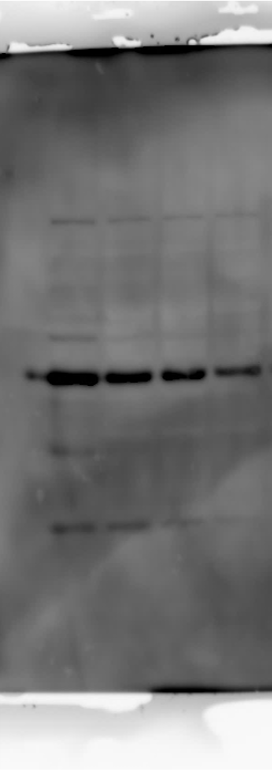

Supplement: Supplementary file 1 [file biomolecules-15-01350-s001.zip › biomolecules-3838184-original-images/Fig.3/Fig.3C. upper panel.tif]

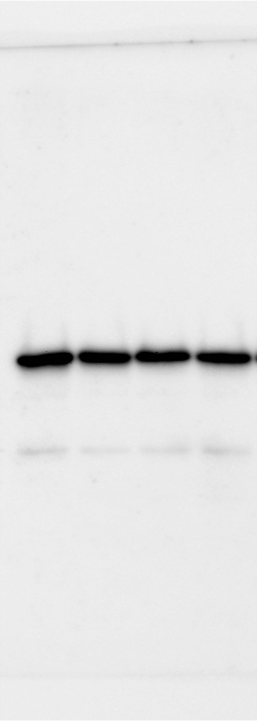

Supplement: Supplementary file 1 [file biomolecules-15-01350-s001.zip › biomolecules-3838184-original-images/Fig.3/Fig.3D. lower panel.tif]

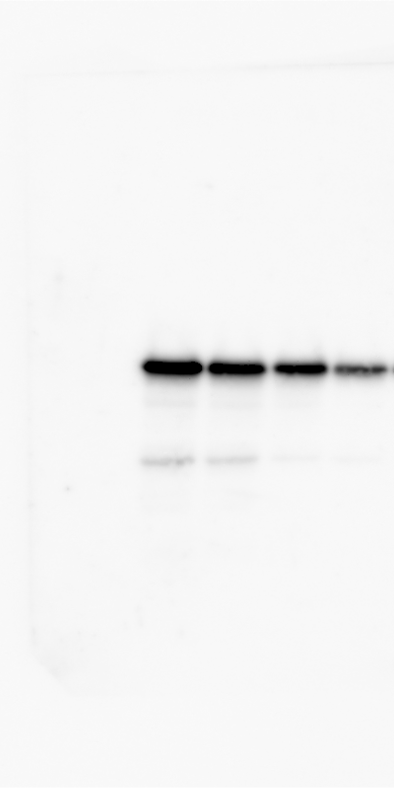

Supplement: Supplementary file 1 [file biomolecules-15-01350-s001.zip › biomolecules-3838184-original-images/Fig.3/Fig.3D. upper panel.tif]

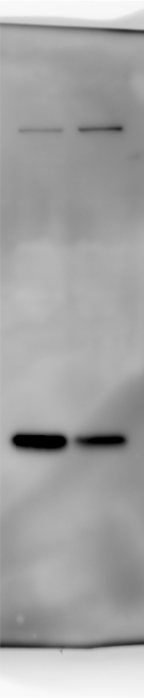

Supplement: Supplementary file 1 [file biomolecules-15-01350-s001.zip › biomolecules-3838184-original-images/Fig.4/Fig.4. 1st and 2nd panel.tif]

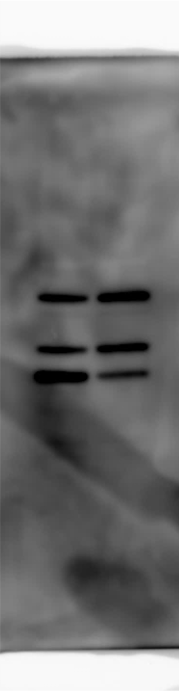

Supplement: Supplementary file 1 [file biomolecules-15-01350-s001.zip › biomolecules-3838184-original-images/Fig.4/Fig.4. 3rd panel.tif]

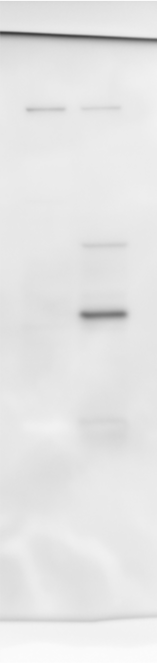

Supplement: Supplementary file 1 [file biomolecules-15-01350-s001.zip › biomolecules-3838184-original-images/Fig.4/Fig.4. 4th panel.tif]

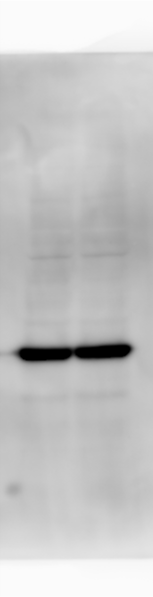

Supplement: Supplementary file 1 [file biomolecules-15-01350-s001.zip › biomolecules-3838184-original-images/Fig.4/Fig.4. 5th panel.tif]
